# Real-world assessment of sparsentan’s drug safety framework

**DOI:** 10.1080/0886022X.2025.2461668

**Published:** 2025-02-19

**Authors:** Wenjing Fu, Jingyu Wang, Yuzhou Xue, Dikang Pan

**Affiliations:** a Department of Nephrology, Mianyang Central Hospital, Mianyang, China; b Renal Division, Peking University First Hospital, Beijing, China; c Department of Cardiology, Peking University Third Hospital, Beijing, China; d Vascular Surgery Department, Xuanwu Hospital, Capital Medical University, Beijing, China

**Keywords:** IgA nephropathy, sparsentan, adverse events, pharmacovigilance, FAERS

## Abstract

**Background:**

Sparsentan has been approved for reducing proteinuria in adult patients with primary IgA nephropathy (IgAN) at risk of rapid disease progression, yet comprehensive studies evaluating its drug safety framework are lacking.

**Methods:**

Adverse event (AE) reports following the market release of sparsentan were collected from the U.S. Food and Drug Administration AE Reporting System. Disproportionate analysis was used to identify previously unrecognized positive novel signals at both the system organ class and preferred term levels. Additionally, analysis on clinical priorities and subgroup analysis were conducted.

**Results:**

A total of 504 patients with IgAN were included. Two novel system organ classes and 14 novel preferred terms were identified. Hypotension and dizziness were established as moderate clinical priority events. Males had a higher relative risk of nausea, peripheral edema, feeling abnormal, decreased blood pressure, and hypotension, while females were at greater risk for fatigue, pain, increased blood creatinine, dizziness, and somnolence. Among those aged 18–45, the relative risk of experiencing fatigue, pain, and dizziness was higher, individuals aged 45 and older had a higher relative risk of peripheral edema, decreased blood pressure, and hypotension.

**Conclusions:**

Based on the available AE reporting data, sparsentan exhibits a favorable safety profile, with no high-priority clinical events identified. Our findings offer valuable insights to optimize the use of sparsentan and understand its potential side effects.

## 1. Introduction

IgA nephropathy (IgAN) is the most common primary glomerulonephritis worldwide and has become a leading cause of chronic kidney disease (CKD) and end-stage renal disease [1]. Recent advances in the therapeutic approach to IgAN have driven by a deeper understanding of the disease’s pathogenesis [2,3]. Alongside an expanding landscape of clinical trials, supported by both financial and corporate backing [4], there has been a marked increase in efforts focusing on both non-immunological and innovative immunological therapies [5–8]. This surge in research is expected to continue in the foreseeable future [9].

Non-immune factors play a critical and indispensable role in the pathogenesis of IgAN. Angiotensin II and endothelin-1 interact with membrane receptors on various renal intrinsic cells, including endothelial cells, epithelial cells, podocytes, and other resident cells, triggering hemodynamic and structural changes within the renal microenvironment. This cascade of events leads to glomerulosclerosis, interstitial fibrosis, interstitial inflammation, and renal tubular atrophy [10–14]. The combined use of renin–angiotensin–aldosterone system inhibitors and endothelin A receptor antagonists has shown cumulative therapeutic benefits [15–18]. Therefore, there is a strong rationale for concurrently targeting these interconnected pathways to optimize the preservation of renal function.

Sparsentan, a dual endothelin and angiotensin II receptor antagonist with high selectivity for the ETA receptor and the angiotensin II subtype 1 receptor, represents a pioneering therapeutic approach for the non-immunologic management of IgAN-associated proteinuria [19]. In February 2023, sparsentan was granted expedited approval in the United States for the reduction of proteinuria in adults with primary IgAN at risk of rapid progression, typically defined by a urine protein/creatinine ratio (UP/C) ≥1.5 g/g [20,21]. This regulatory milestone was primarily based on interim data from the PROTECT phase III trial [22]. Analysis demonstrated that, after 36 weeks of treatment, patients receiving sparsentan experienced a significantly greater reduction in proteinuria from baseline compared to the control group, who were treated with irbesartan (49.8% vs. 15.1%, *p* < .0001). Post hoc sensitivity analysis, requested by the U.S. Food and Drug Administration (FDA), also confirmed these results [23].

The complete data from the PROTECT phase III trial have been published [24]. Compared to the irbesartan group, sparsentan demonstrated remarkable renal function preservation and a significant reduction in proteinuria. Specifically, sparsentan induced a rapid and sustained decrease in proteinuria over 110 weeks, with a 40% lower UP/C, and 31% of patients achieving complete remission of proteinuria (UP/C < 0.3), compared to only 11% in the control group. Additionally, the rate of decline in estimated glomerular filtration rate was lower in the sparsentan group compared to the control group (−2.7 vs. −3.8 mL/min/1.73 m^2^). Furthermore, the incidence of adverse events (AEs) was comparable between the two groups. Given the anticipated widespread clinical use of sparsentan, it will be essential to further utilize real-world data reporting systems to monitor its post-marketing safety.

In this study, we utilized the FDA Adverse Event Reporting System (FAERS) database, the largest AE reporting database globally [25], to extract reports related to sparsentan. Through disproportionate analysis, we conducted a comprehensive assessment of the safety profile and potential risks associated with sparsentan, with the goal of providing guidance and ensuring vigilance for its rational clinical use.

## 2. Methods

### 2.1. Data collection and processing

AEs in the FAERS database are reported voluntarily by healthcare professionals, manufacturers, and consumers. The system comprises seven datasets, including patient demographic and administrative information, drug and biological particulars, AEs, patient outcomes, report origins, drug therapy initiation and conclusion dates, and utilization/diagnostic indications. For the purpose of this analysis, only the most severe outcome for each individual was documented. Death takes precedence over life-threatening, which in turn take precedence over disability, hospitalization, other important medical events (IMEs), and unknown outcomes.

A safety analysis of sparsentan post-market launch in IgAN patients was conducted using data from the first to the fourth quarter of 2023. Information retrieval was achieved by combining the generic name (sparsentan), brand name (Filspari), and alternative names (DEARA, PS-433540, RE-021) of the medication [21]. The FAERS database categories drug effects into four groups: primary suspect (PS), secondary suspect, concomitant, and interacting. To refine analytical precision and reduce confounding factors, only reports where sparsentan was identified as the PS drug in the AE were included. Data deduplication was performed following FDA-recommended guidelines [26,27], which involve: (i) selecting the most recent FDA_DT if CASEIDs match and (ii) prioritizing the higher PRIMARYID when both CASEID and FDA_DT are identical.

**Figure 1.**
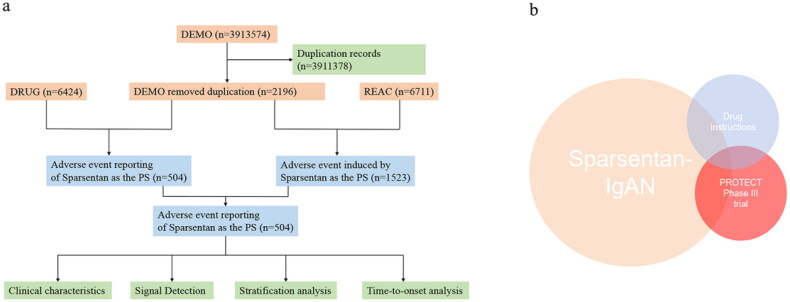
Data processing procedures. (a) The process of screening sparsentan-associated AEs from FAERS. (b) Novel AEs associated with sparsentan were screened by excluding intersections. DEMO: the file descriptor for demographic and administrative information; DRUG: the file descriptor for drug and biological details; REAC: the file descriptor for adverse events; PS: primary suspect; AEs: adverse events.

After excluding duplicate reports, the final dataset comprised 2,196 unique reports, with 504 instances of sparsentan identified as the PS in the IgAN cohort. Figure 1 illustrates the data processing workflow.

### 2.2. Disproportionate analysis and signal detection

Disproportionate analysis, based on a 2 × 2 contingency table, is used to assess the potential correlation between AEs and specific medications [28]. A positive signal for a medication emerges when the incidence rate of a particular AE significantly exceeds the background frequency within the database and surpasses a predefined threshold [26,29].

In this study, we applied two disproportionate analysis algorithms: the reporting odds ratio (ROR) and the proportional reporting ratio (PRR), for signal exploration. Detailed formulas and signal detection thresholds are provided in Supplementary Table 1. To accurately identify novel potential AEs for sparsentan in the IgAN population, we implemented the following exclusion criteria: (i) preferred terms associated closely with IgAN progression were excluded, (ii) preferred terms with erroneous or ambiguous usage were omitted, and (iii) previously reported AEs, as referenced in drug inserts (Supplementary Table 2) and the PROTECT phase III trial (Supplementary Table 3), were excluded. These procedures, designed to eliminate duplicates, synonymous, and near-synonymous preferred terms, were guided by the Medical Dictionary for Regulatory Activities, version 25.0.

### 2.3. Clinical priority analysis

To assess the clinical priority of positive preferred terms, we evaluated them using five indicators: the number of target events, the lower limit of ROR, the proportion of mortality, adherence to IMEs or designated medical events (DMEs) criteria, and biological plausibility (Table 1) [30]. IMEs and DMEs, defined and standardized by the EMA, capture varying levels of severity and drug-attributable risk [31,32]. IMEs refer to AEs with well-defined severity characteristics, while DMEs identify rare, severe AEs with a high drug-attributable risk, which may raise safety concerns in specific contexts. Each dimension is categorized into three levels, with corresponding scores of 0, 1, or 2. When an event meets multiple criteria within a dimension, the highest score is applied to ensure maximal attribution. AEs accumulating scores within the ranges of 0–4, 5–7, or 8–10 are classified as weak, moderate, or strong clinical priorities, respectively.

**Table 1.** A rating scale assessing clinical priority of disproportionality signals.

Assessment items	2 points	1 point	0 point
Number of target events (a)	>50	10–50	<10
Lower limit of ROR	>5	2–5	1–2
Mortality proportion	>50%	25–50%	<25%
IMEs or DMEs	DME	IME	None
Biological plausibility	Recognized as AE in the drug inserts	Recognized as AE in the PROTECT phase III trial	Lack of clear reporting or documentation

Mortality proportion: percentage of cases in which death was reported as an outcome in the overall cases report for a particular AE. IMEs and DMEs are developed and updated by EMA.

### 2.4. Subgroup analysis

Subgroup analysis was conducted based on age (with 45 years as the conventional threshold distinguishing youth from middle-aged/elderly individuals) and gender to strengthen the association between sparsentan and AEs, while minimizing the influence of demographic variables on study outcomes.

### 2.5. Statistics

Disproportionate analysis was employed to assess the association between AEs and sparsentan, with the corresponding algorithmic standards detailed in Supplementary Table 1. Data processing, visualization, and statistical analysis were conducted using R (version 4.3.2). This study adheres to the Strengthening the Reporting of Observational Studies in Epidemiology guidelines.

## 3. Results

### 3.1. General characteristics in the real-world population

A total of 504 patients were included, all of whom were from the United States (Table 2). Males represented a slightly higher proportion of reports (55%) compared to females (43.5%). The middle-aged and elderly group (aged 46 years and older) constituted the largest proportion of those reporting AEs at 40.67%, followed by the youthful cohort (aged 18–45 years) at 33.33%. Among the limited known outcomes, ‘Other important medical events’ accounted for 6.2%.

**Table 2.** Clinical characteristics of patients treated for IgAN with sparsentan in the FAERS Database.

Characteristics	Case number, *n*	Case proportion, %
Gender		
Female	219	43.50%
Male	277	55.00%
Unknown	8	1.60%
Age		
<18 years	2	0.40%
18–45 years	168	33.33%
>46 years	205	40.67%
Unknown	129	25.60%
Outcome		
Hospitalization	13	2.60%
Other important medical event	31	6.20%
Unknown	460	91.30%
Reported countries		
USA	504	100.00%
Reporting year		
2023	504	100.00%

### 3.2. Positive system organ classes

A total of 24 system organ classes were implicated (Table 3). Only nervous system disorders (228 reports) and ear and labyrinth disorders (nine reports) met the criteria for positive signals according to the disproportionate analysis algorithms. Furthermore, general disorders and administration site conditions (338 reports), gastrointestinal disorders (188 reports), and respiratory, thoracic, and mediastinal disorders (62 reports) met the criteria of the ROR algorithm.

**Table 3.** Signal detection results at the level of system organ classes.

System organ classes	a	b	c	d	ROR (95%CI)	PRR (*χ*^2^)
General disorders and administration site conditions^#^	338	1185	971	4217	1.24 (1.08–1.42)	1.19 (9.06)
Nervous system disorders^*^	228	1295	356	4832	2.39 (2–2.85)	2.18 (97.43)
Investigations	195	1328	603	4585	1.12 (0.94–1.33)	1.1 (1.57)
Gastrointestinal disorders^#^	188	1335	481	4707	1.38 (1.15–1.65)	1.33 (12.39)
Musculoskeletal and connective tissue disorders	99	1424	326	4862	1.04 (0.82–1.31)	1.03 (0.09)
Injury, poisoning and procedural complications	77	1446	494	4694	0.51 (0.4–0.65)	0.53 (30.17)
Respiratory, thoracic and mediastinal disorders^#^	62	1461	149	5039	1.44 (1.06–1.94)	1.42 (5.56)
Renal and urinary disorders	59	1464	252	4936	0.79 (0.59–1.05)	0.8 (2.58)
Vascular disorders	52	1471	204	4984	0.86 (0.63–1.18)	0.87 (0.86)
Infections and infestations	51	1472	266	4922	0.64 (0.47–0.87)	0.65 (8.28)
Skin and subcutaneous tissue disorders	48	1475	271	4917	0.59 (0.43–0.81)	0.6 (11.16)
Psychiatric disorders	32	1491	233	4955	0.46 (0.31–0.66)	0.47 (17.73)
Metabolism and nutrition disorders	27	1496	133	5055	0.69 (0.45–1.04)	0.69 (3.16)
Cardiac disorders	13	1510	39	5149	1.14 (0.61–2.13)	1.14 (0.16)
Eye disorders	12	1511	82	5106	0.49 (0.27–0.91)	0.5 (5.36)
Blood and lymphatic system disorders	10	1513	24	5164	1.42 (0.68–2.98)	1.42 (0.88)
Ear and labyrinth disorders^*^	9	1514	7	5181	4.4 (1.64–11.83)	4.38 (10.29)
Immune system disorders	7	1516	37	5151	0.64 (0.29–1.44)	0.64 (1.16)
Hepatobiliary disorders	5	1518	30	5158	0.57 (0.22–1.46)	0.57 (1.42)
Surgical and medical procedures	4	1519	103	5085	0.13 (0.05–0.35)	0.13 (22.27)
Reproductive system and breast disorders	4	1519	49	5139	0.28 (0.1–0.77)	0.28 (6.99)
Endocrine disorders	1	1522	48	5140	0.07 (0.01–0.51)	0.07 (12)
Social circumstances	1	1522	5	5183	0.68 (0.08–5.83)	0.68 (0.12)
Product issues	1	1522	4	5184	0.85 (0.1–7.62)	0.85 (0.02)

^*^ Meets criteria for both algorithm thresholds.

^#^ Meets criteria for one algorithm threshold.

### 3.3. Positive preferred terms

**Figure 2.**
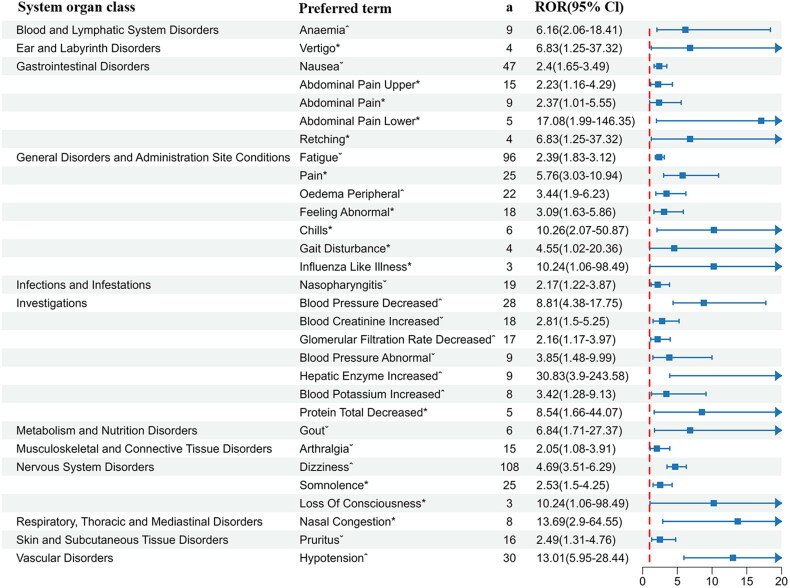
The forest plot of the 30 positive preferred terms associated with sparsentan. ˆRecognized as AE in the drug inserts. ˇRecognized as AE in the PROTECT phase III trial. *Novel signals. ^a^Number of reports containing both the target drug and target adverse drug reaction. ROR: reporting odds ratio.

A total of 30 positive preferred terms, spanning 12 system organ classes, were identified, with ‘hepatic enzyme increased’ emerging as the most prominent signal (ROR 30.83, PRR 30.66) (Figure 2, Table 4). After the exclusion procedures outlined earlier, 14 novel AEs, previously unreported, emerged across six system organ classes, with pain (25 reports) and somnolence (25 reports) being the most frequently documented.

**Table 4.** Thirty PTs that meet the criteria of the two algorithms for sparsentan.

System organ classes	PT	a	b	c	d	ROR (95%CI)	PRR (*χ*^2^)
Blood and lymphatic system disorders	Anemia^ˆ^	9	1514	5	5183	6.16 (2.06–18.41)	6.13 (13.83)
Ear and labyrinth disorders	Vertigo^*^	4	1519	2	5186	6.83 (1.25–37.32)	6.81 (6.62)
Gastrointestinal disorders	Nausea^ˇ^	47	1476	68	5120	2.4 (1.65–3.49)	2.35 (22.03)
	Abdominal pain (upper part)^*^	15	1508	23	5165	2.23 (1.16–4.29)	2.22 (6.13)
	Abdominal pain^*^	9	1514	13	5175	2.37 (1.01–5.55)	2.36 (4.17)
	Abdominal pain (lower part)^*^	5	1518	1	5187	17.08 (1.99–146.35)	17.03 (12.59)
	Retching^*^	4	1519	2	5186	6.83 (1.25–37.32)	6.81 (6.62)
General disorders and administration site conditions	Fatigue^ˇ^	96	1427	142	5046	2.39 (1.83–3.12)	2.3 (43.78)
	Pain^*^	25	1498	15	5173	5.76 (3.03–10.94)	5.68 (36.34)
	Edema peripheral^ˆ^	22	1501	22	5166	3.44 (1.9–6.23)	3.41 (18.82)
	Feeling abnormal^*^	18	1505	20	5168	3.09 (1.63–5.86)	3.07 (13.26)
	Chills^*^	6	1517	2	5186	10.26 (2.07–50.87)	10.22 (12.49)
	Gait disturbance^*^	4	1519	3	5185	4.55 (1.02–20.36)	4.54 (4.74)
	Influenza like illness^*^	3	1520	1	5187	10.24 (1.06–98.49)	10.22 (6.24)
Infections and infestations	Nasopharyngitis^ˇ^	19	1504	30	5158	2.17 (1.22–3.87)	2.16 (7.28)
Investigations	Blood pressure decreased^ˆ^	28	1495	11	5177	8.81 (4.38–17.75)	8.67 (53.91)
	Blood creatinine increased^ˇ^	18	1505	22	5166	2.81 (1.5–5.25)	2.79 (11.41)
	Glomerular filtration rate decreased^ˆ^	17	1506	27	5161	2.16 (1.17–3.97)	2.14 (6.42)
	Blood pressure abnormal^ˇ^	9	1514	8	5180	3.85 (1.48–9.99)	3.83 (8.89)
	Hepatic enzyme increased^ˆ^	9	1514	1	5187	30.83 (3.9–243.58)	30.66 (25.86)
	Blood potassium increased^ˆ^	8	1515	8	5180	3.42 (1.28–9.13)	3.41 (6.82)
	Protein total decreased^*^	5	1518	2	5186	8.54 (1.66–44.07)	8.52 (9.49)
Metabolism and nutrition disorders	Gout^ˇ^	6	1517	3	5185	6.84 (1.71–27.37)	6.81 (9.93)
Musculoskeletal and connective tissue disorders	Arthralgia^ˇ^	15	1508	25	5163	2.05 (1.08–3.91)	2.04 (5.03)
Nervous system disorders	Dizziness^ˆ^	108	1415	83	5105	4.69 (3.51–6.29)	4.43 (128.4)
	Somnolence^*^	25	1498	34	5154	2.53 (1.5–4.25)	2.5 (13.14)
	Loss of consciousness^*^	3	1520	1	5187	10.24 (1.06–98.49)	10.22 (6.24)
Respiratory, thoracic and mediastinal disorders	Nasal congestion^*^	8	1515	2	5186	13.69 (2.9–64.55)	13.63 (18.75)
Skin and subcutaneous tissue disorders	Pruritus^ˇ^	16	1507	22	5166	2.49 (1.31–4.76)	2.48 (8.21)
Vascular disorders	Hypotension^ˆ^	30	1493	8	5180	13.01 (5.95–28.44)	12.77 (68.93)

^*^ Novel signals.

^ˆ^ Recognized as AE in the drug inserts.

^ˇ^ Recognized as AE in the PROTECT phase III trial.

### 3.4. Clinical priority

Among the 30 positive signals examined, no instances of mortality were reported. Only ‘loss of consciousness’ was classified within the IME category. After comprehensive scoring, 28 events were categorized as low clinical priority, while two events – ‘hypotension’ and ‘dizziness’ – were considered of moderate clinical priority (Table 5).

**Table 5.** Clinical priority assessing results of disproportionality signals.

Preferred terms	Number of target events (score)	Lower limit of ROR (score)	Death (score)	IMEs or DMEs (score)	Biological plausibility (score)	Priority level (score)
Influenza like illness	0	0	0	0	0	Low (0)
Gait disturbance	0	0	0	0	0	Low (0)
Vertigo	0	0	0	0	0	Low (0)
Retching	0	0	0	0	0	Low (0)
Protein total decreased	0	0	0	0	0	Low (0)
Abdominal pain (lower part)	0	0	0	0	0	Low (0)
Abdominal pain	0	0	0	0	0	Low (0)
Loss of consciousness	0	0	0	1	0	Low (1)
Gout	0	0	0	0	1	Low (1)
Chills	0	1	0	0	0	Low (1)
Nasal congestion	0	1	0	0	0	Low (1)
Blood pressure abnormal	0	0	0	0	1	Low (1)
Abdominal pain (upper part)	1	0	0	0	0	Low (1)
Feeling abnormal	1	0	0	0	0	Low (1)
Somnolence	1	0	0	0	0	Low (1)
Blood potassium increased	0	0	0	0	2	Low (2)
Arthralgia	1	0	0	0	1	Low (2)
Pruritus	1	0	0	0	1	Low (2)
Blood creatinine increased	1	0	0	0	1	Low (2)
Nasopharyngitis	1	0	0	0	1	Low (2)
Pain	1	1	0	0	0	Low (2)
Nausea	1	0	0	0	1	Low (2)
Anemia	0	1	0	0	2	Low (3)
Hepatic enzyme increased	0	1	0	0	2	Low (3)
Glomerular filtration rate decreased	1	0	0	0	2	Low (3)
Edema peripheral	1	0	0	0	2	Low (3)
Fatigue	2	0	0	0	1	Low (3)
Blood pressure decreased	1	1	0	0	2	Low (4)
Hypotension	1	2	0	0	2	Moderate (5)
Dizziness	2	1	0	0	2	Moderate (5)

### 3.5. Subgroup analysis results

**Figure 3.**
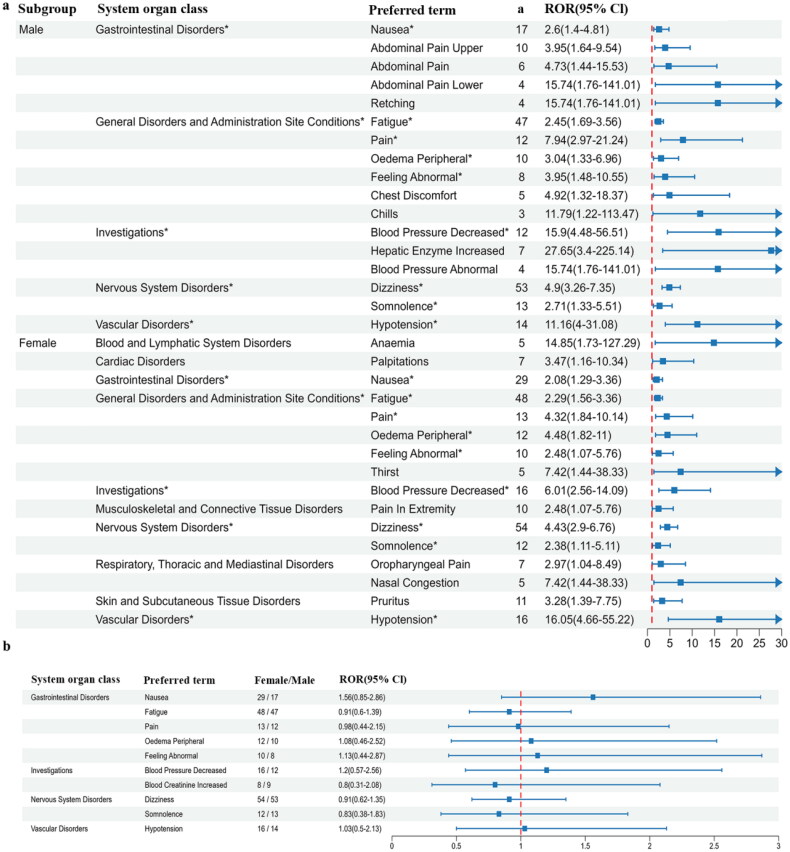
Forest plot depicting AEs in gender subgroups. (a) All positive preferred terms. *Preferred terms shared by both male and female cohorts. (b) Relative risk of shared preferred terms for different gender subgroups. ^a^Number of reports containing both the target drug and target adverse drug reaction. ROR: reporting odds ratio.

In the male subgroup, a total of 17 positive signals were identified, compared to 16 in females (Figure 3(a), Supplementary Table 4). The most frequently reported signal by both genders was dizziness (males: 53 reports, females: 54 reports). The signals with the highest intensity for each gender were ‘hepatic enzyme increased’ (males: ROR 27.65, PRR 27.39) and ‘hypotension’ (females: ROR 16.05, PRR 15.74). Further extraction of positive preferred terms co-reported by both genders was subjected to disproportionate analysis. Males exhibited a higher or slightly higher relative risk than females for nausea, peripheral edema, abnormal feeling, decreased blood pressure, and hypotension, while females showed slightly higher rates for fatigue, pain, increased blood creatinine, dizziness, and somnolence (Figure 3(b)).

**Figure 4.**
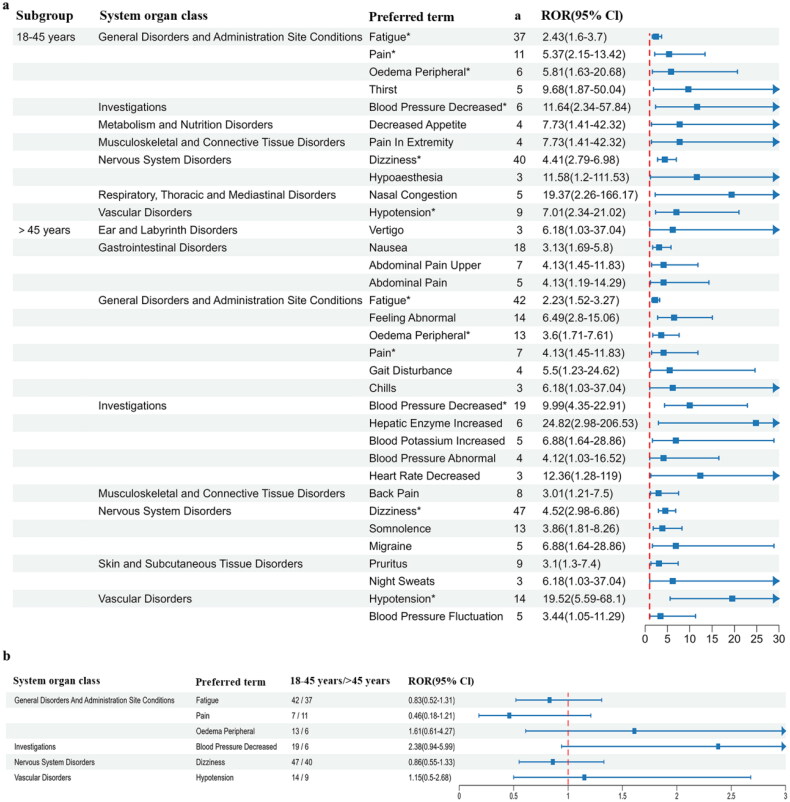
Forest plot depicting AEs in age subgroups. (a) All positive preferred terms. *Preferred terms shared by both age subgroups. (b) Relative risk of shared preferred terms for different age subgroups. ^a^Number of reports containing both the target drug and target adverse drug reaction. ROR: reporting odds ratio.

In the young to young-middle aged cohort, a total of 11 positive signals were identified, compared to 23 in middle-aged/elderly cohort (Figure 4(a), Supplementary Table 5). The most frequently reported signal by both subgroup was dizziness (18–45 years: 40 reports, >45 years: 47 reports). The signals with the highest intensity for each age subgroup were ‘nasal congestion’ (18–45 years: ROR 19.37, PRR 19.19) and ‘hepatic enzyme increased’ (>45 years: ROR 24.82, PRR 24.62). Further extraction of positive preferred terms co-reported by both subgroup was subjected to disproportionate analysis. In relative terms, the 18–45 age group exhibits a higher risk of experiencing fatigue, pain, and dizziness, whereas those aged 45 and above showed a heightened occurrence of peripheral edema, decreased blood pressure, and hypotension (Figure 4(b)).

## 4. Discussion

The higher reporting rate of AEs in males compared to females (Table 2), based on reports from the United States may reflect the gender disparity in the incidence of IgAN (with a male-to-female ratio of 3:1) in the North American population [33]. Among the reported outcomes, sparsentan demonstrated a favorable safety profile, with no cases of death, life-threatening events, or disabilities recorded across the 504 reported instances.

To date, there is a notable lack of searchable or accessible records of system organ classes related to sparsentan, both in the IgAN and focal segmental glomerulosclerosis (FSGS) cohorts. Through disproportionate analysis, we identified two positive system organ classes: nervous system disorders and ear and labyrinth disorders (Table 3). Within these categories, three AEs were reported: vertigo (ROR 6.83, PRR 9.81), somnolence (ROR 2.53, PRR 2.5), and loss of consciousness (ROR 10.24, PRR 10.22) (Table 4). Notably, loss of consciousness emerged as the most severe among the 14 novel AEs. Consequently, we conducted a deeper investigation into the individuals reporting loss of consciousness and reviewed their preferred terms (Supplementary Table 6). These patients, diagnosed with IgAN, CKD, and proteinuria, did not present with other conditions commonly associated with consciousness disturbances, such as severe infections, cerebrovascular accidents, endocrine metabolic disorders, poisoning, physical or hypoxic damage, or sleep deprivation. However, intriguingly, all these individuals reported issues with drug intolerance or product dose omission. Sparsentan exhibits pharmacokinetic characteristics consistent with a two-compartment model, with dose-dependent bioavailability being a key feature [21,34]. As such, fluctuations in drug concentrations in the body are inevitable, potentially increasing the risk of atypical outcomes. In other words, in the absence of regular dosing or drug intolerance, the risk of loss of consciousness may be reduced. Unfortunately, the limited number of available cases restricts our ability to perform correlation analysis.

Significantly, we identified 11 additional new AEs, including abdominal pain, retching, pain, feeling abnormal, chills, gait disturbance, influenza like illness, protein total decreased, and nasal congestion. The PROTECT phase III trial also documented gastroesophageal reflux disease, diarrhea, nausea, and nasopharyngitis, consistent with our findings. However, according to the scrutiny of Medical Dictionary for Regulatory Activities, these events are independent and not synonymous preferred terms. Therefore, continuous monitoring of these events is essential during sparsentan therapy. The DUPLEX trial assessed the safety of sparsentan in 184 patients with FSGS [35]. Common AEs, reported by more than 10% of patients, included anemia, back pain, increased blood creatine kinase levels, CKD, COVID-19, diarrhea, dizziness, headache, hyperkalemia, hypertension, hypotension, muscle spasms, nausea, and peripheral edema. Notably, anemia, hypotension, dizziness, and nausea overlapped with findings in this study. Further research is needed to explore the potential common mechanistic basis underlying these AEs associated with sparsentan.

The clinical priority assessment provides valuable insights into the management of potential AEs and underscores the favorable safety profile of sparsentan. Among the 30 positive preferred terms, no events were classified as highly clinically prioritized, with only hypotension and dizziness being identified as moderately clinically prioritized. A thorough review of these 30 preferred terms indicates that appropriate monitoring or follow-up is warranted for liver and kidney function, hematological parameters, blood pressure, concomitant medications, and physical symptoms. Management strategies for the potential risk of hypotension are detailed in the label [20]. First, maintaining adequate volume status is recommended; second, the necessity of adjusting antihypertensive medications should be evaluated. If hypotension persists despite discontinuing or reduction of other antihypertensive agents, a dose reduction or interruption of sparsentan may be considered. Transient, self-limiting hypotensive reactions do not constitute a contraindication for continuing sparsentan therapy, and treatment can be resumed once blood pressure stabilizes. In summary, the drug label thoroughly addresses moderately clinically prioritized AEs, while our findings effectively supplement inadequately covered low clinically prioritized events, emphasizing the need for ongoing monitoring and vigilance, especially for new signals, in the future.

Subgroup analysis provides insights into the potential differential occurrence of AEs across populations, offering valuable guidance for the precision prevention of AEs in specific groups. For instance, older adults exhibit a higher relative risk of peripheral edema, decreased blood pressure, and hypotension, likely due to increased susceptibility to vascular sclerosis, circulatory failure, and orthostatic hypotension [36–39]. In contrast, younger individuals are more prone to fatigue and dizziness, with factors such as inadequate sleep, overwork, and high workloads potentially contributing [40–42]. As the population receiving sparsentan expands, prospective data from large cohorts will be essential to elucidate the underlying mechanisms driving these differential risks.

Naturally, our study has several inherent limitations. First, as a spontaneous reporting system, FAERS is prone to biases inherent in voluntary reporting, and reliance solely on disproportionate analysis algorithms may not provide sufficient information to accurately assess the true risk of AE occurrence. Consequently, high signal values for AEs do not necessarily correlate with a heightened risk in clinical practice. Second, FAERS cannot establish causality between drugs and AEs, nor can it calculate the incidence rate of AEs. Our findings can only suggest a potential association between sparsentan and specific AEs, which should be regarded as hypothesis-generating rather than conclusive. Third, although our study was designed to track AEs of sparsentan for pharmacovigilance purposes, its relatively short time on the market means that treatment durations are often limited. Furthermore, early adopters may not fully represent the typical patient population managed by nephrologists. As prescribing volumes increase, the current AE data may not fully reflect the broader community.

## 5. Conclusions

This study is the first to identify potential AEs associated with sparsentan using the FAERS database. Our findings demonstrate the favorable safety profile of sparsentan while highlighting the importance of early clinical vigilance, hematologic monitoring, and careful attention to potential AEs. Given the inherent biases of spontaneous reporting systems, further cohort studies and long-term data are required to validate these findings.

## Supplementary Material

Supplemental Material

## Data Availability

Publicly available datasets were analyzed in this study. This data can be found here: All data come from the FAERS database, which is available at https://fis.fda.gov/extensions/FPD-QDE-FAERS/FPD-QDE-FAERS.html.
