# Supplementary material for: Real-world assessment of sparsentan’s drug safety framework
Source: Ren Fail. 2025 Feb 19;47(1):2461668. doi: 10.1080/0886022X.2025.2461668 (PMC11843636; doi:10.1080/0886022X.2025.2461668)
Supplement: Supplemental Material [file IRNF_A_2461668_SM9748.docx]

**Supplementary Table 1** DPA algorithms for signal detection at levels of system organ classes and preferred terms.

| Algorithms | Equation | Criteria |
| --- | --- | --- |
| ROR | ROR=ad/b/c | Lower limit of 95% CI>1, a≥3 |
|  | 95%CI=e^ln(ROR)±1.96(1/a+1/b+1/c+1/d)^0.5^ |  |
| PRR | PRR=a(c+d)/c/(a+b) | PRR≥2, χ^2^≥4, a≥3 |
|  | χ^2^=[(ad-bc)^2](a+b+c+d)/[(a+b)(c+d)(a+c)(b+d)] |  |

Equation: a, number of reports containing both the target drug and target adverse drug reaction; b, number of reports containing other adverse drug reaction of the target drug; c, number of reports containing the target adverse drug reaction of other drugs; d, number of reports containing other drugs and other adverse drug reactions. 95%CI, 95% confidence interval; N, the number of reports; χ^2^, chi-squared.

**Supplementary Table 2** Overview of sparsentan approved by FDA.

| Target | Brand name | Approval or authorisation date | Officially approved organization | Link |
| --- | --- | --- | --- | --- |
| Sparsentan | [Filspari](https://www.ema.europa.eu/en/medicines/human/EPAR/filspari) | 02/17/2023 | FDA | <https://www.accessdata.fda.gov/drugsatfda_docs/label/2023/216403s000lbl.pdf> |

Supplementary Table 3. AEs disclosed in the PROTECT phase III trial.

| Categories | AEs |
| --- | --- |
| Treatment-emergent AEs | COVID-19 |
|  | Hyperkalaemia |
|  | Peripheral Oedema |
|  | Dizziness |
|  | Headache |
|  | Hypotension |
|  | Hypertension |
|  | Upper Respiratory Tract Infection |
|  | Fatigue |
|  | Anaemia |
|  | Nasopharyngitis |
|  | Blood Creatine Phosphokinase Increased |
|  | Cough |
|  | Muscle Spasms |
|  | Arthralgia |
|  | Proteinuria |
|  | Back Pain |
|  | Lipase Increased |
|  | Acute Kidney Injury |
|  | Gout |
|  | Pruritus |
|  | Diarrhoea |
|  | Blood Creatinine Increased |
|  | Alanine Aminotransferase Increased |
|  | Gastro-Oesophageal Reflux Disease |
|  | Nausea |
|  | Myalgia |
|  | Renal Impairment |
|  | Urinary Tract Infection |
|  | Hyperuricaemia |
|  | Pain In Extremity |
|  | Aminotransferase Elevations |
| Serious treatment-emergent AEs | COVID-19 |
|  | Chronic Kidney Disease |
|  | Acute Kidney Injury |
|  | Dizziness |
|  | Proteinuria |
|  | Malaise |
|  | Appendicitis |
|  | Cellulitis |
|  | Covid-19 Pneumonia |
|  | Iga Nephropathy |
|  | Meniscus Injury |
| Treatment-emergent AEs leading to treatment discontinuation | Chronic Kidney Disease |
|  | Acute Kidney Injury |
|  | Alanine Aminotransferase Increased |
|  | Hypotension |
|  | Lipase Increased |
|  | Renal Impairment |

**Supplementary Table 4** PTs data for gender subpopulations. *Denotes preferred terms shared by both age cohorts.

| Subgroup | System Organ Classes | Preferred Terms | a | b | c | d | ROR(95%Cl) | PRR(χ2) |
| --- | --- | --- | --- | --- | --- | --- | --- | --- |
| Male | Gastrointestinal Disorders | Nausea* | 17 | 698 | 26 | 2772 | 2.6 (1.4 - 4.81) | 2.56 (9.88) |
|  |  | Abdominal Pain Upper | 10 | 705 | 10 | 2788 | 3.95 (1.64 - 9.54) | 3.91 (10.91) |
|  |  | Abdominal Pain | 6 | 709 | 5 | 2793 | 4.73 (1.44 - 15.53) | 4.7 (7.96) |
|  |  | Abdominal Pain Lower | 4 | 711 | 1 | 2797 | 15.74 (1.76 - 141.01) | 15.65 (10.99) |
|  |  | Retching | 4 | 711 | 1 | 2797 | 15.74 (1.76 - 141.01) | 15.65 (10.99) |
|  | General Disorders and Administration Site Conditions | Fatigue* | 47 | 668 | 78 | 2720 | 2.45 (1.69 - 3.56) | 2.36 (23.78) |
|  |  | Pain* | 12 | 703 | 6 | 2792 | 7.94 (2.97 - 21.24) | 7.83 (23.94) |
|  |  | Oedema Peripheral* | 10 | 705 | 13 | 2785 | 3.04 (1.33 - 6.96) | 3.01 (7.64) |
|  |  | Feeling Abnormal* | 8 | 707 | 8 | 2790 | 3.95 (1.48 - 10.55) | 3.91 (8.71) |
|  |  | Chest Discomfort | 5 | 710 | 4 | 2794 | 4.92 (1.32 - 18.37) | 4.89 (6.9) |
|  |  | Chills | 3 | 712 | 1 | 2797 | 11.79 (1.22 - 113.47) | 11.74 (7.38) |
|  | Investigations | Blood Pressure Decreased* | 12 | 703 | 3 | 2795 | 15.9 (4.48 - 56.51) | 15.65 (33.06) |
|  |  | Hepatic Enzyme Increased | 7 | 708 | 1 | 2797 | 27.65 (3.4 - 225.14) | 27.39 (22.3) |
|  |  | Blood Pressure Abnormal | 4 | 711 | 1 | 2797 | 15.74 (1.76 - 141.01) | 15.65 (10.99) |
|  | Nervous System Disorders | Dizziness* | 53 | 662 | 45 | 2753 | 4.9 (3.26 - 7.35) | 4.61 (70.75) |
|  |  | Somnolence* | 13 | 702 | 19 | 2779 | 2.71 (1.33 - 5.51) | 2.68 (8.19) |
|  | Vascular Disorders | Hypotension* | 14 | 701 | 5 | 2793 | 11.16 (4 - 31.08) | 10.96 (33.52) |
| Female | Blood and Lymphatic System Disorders | Anaemia | 5 | 789 | 1 | 2343 | 14.85 (1.73 - 127.29) | 14.76 (10.71) |
|  | Cardiac Disorders | Palpitations | 7 | 787 | 6 | 2338 | 3.47 (1.16 - 10.34) | 3.44 (5.63) |
|  | Gastrointestinal Disorders | Nausea* | 29 | 765 | 42 | 2302 | 2.08 (1.29 - 3.36) | 2.04 (9.28) |
|  | General Disorders and Administration Site Conditions | Fatigue* | 48 | 746 | 64 | 2280 | 2.29 (1.56 - 3.36) | 2.21 (18.94) |
|  |  | Pain* | 13 | 781 | 9 | 2335 | 4.32 (1.84 - 10.14) | 4.26 (13.38) |
|  |  | Oedema Peripheral* | 12 | 782 | 8 | 2336 | 4.48 (1.82 - 11) | 4.43 (12.82) |
|  |  | Feeling Abnormal* | 10 | 784 | 12 | 2332 | 2.48 (1.07 - 5.76) | 2.46 (4.76) |
|  |  | Thirst | 5 | 789 | 2 | 2342 | 7.42 (1.44 - 38.33) | 7.38 (7.9) |
|  | Investigations | Blood Pressure Decreased* | 16 | 778 | 8 | 2336 | 6.01 (2.56 - 14.09) | 5.9 (21.89) |
|  | Musculoskeletal and Connective Tissue Disorders | Pain In Extremity | 10 | 784 | 12 | 2332 | 2.48 (1.07 - 5.76) | 2.46 (4.76) |
|  | Nervous System Disorders | Dizziness* | 54 | 740 | 38 | 2306 | 4.43 (2.9 - 6.76) | 4.2 (55.92) |
|  |  | Somnolence* | 12 | 782 | 15 | 2329 | 2.38 (1.11 - 5.11) | 2.36 (5.28) |
|  | Respiratory, Thoracic and Mediastinal Disorders | Oropharyngeal Pain | 7 | 787 | 7 | 2337 | 2.97 (1.04 - 8.49) | 2.95 (4.54) |
|  |  | Nasal Congestion | 5 | 789 | 2 | 2342 | 7.42 (1.44 - 38.33) | 7.38 (7.9) |
|  | Skin and Subcutaneous Tissue Disorders | Pruritus | 11 | 783 | 10 | 2334 | 3.28 (1.39 - 7.75) | 3.25 (8.2) |
|  | Vascular Disorders | Hypotension* | 16 | 778 | 3 | 2341 | 16.05 (4.66 - 55.22) | 15.74 (35.1) |

**Supplementary Table 5** PTs data for age subpopulations. *Denotes preferred terms shared by both age cohorts.

| Subgroup | System Organ Classes | Preferred Terms | a | b | c | d | ROR(95%Cl) | PRR(χ2) |
| --- | --- | --- | --- | --- | --- | --- | --- | --- |
| 18-45 years | General Disorders and Administration Site Conditions | Fatigue* | 37 | 484 | 61 | 1939 | 2.43 (1.6 - 3.7) | 2.33 (18.16) |
|  |  | Pain* | 11 | 510 | 8 | 1992 | 5.37 (2.15 - 13.42) | 5.28 (16.18) |
|  |  | Oedema Peripheral* | 6 | 515 | 4 | 1996 | 5.81 (1.63 - 20.68) | 5.76 (9.47) |
|  |  | Thirst | 5 | 516 | 2 | 1998 | 9.68 (1.87 - 50.04) | 9.6 (11.03) |
|  | Investigations | Blood Pressure Decreased* | 6 | 515 | 2 | 1998 | 11.64 (2.34 - 57.84) | 11.52 (14.45) |
|  | Metabolism and Nutrition Disorders | Decreased Appetite | 4 | 517 | 2 | 1998 | 7.73 (1.41 - 42.32) | 7.68 (7.76) |
|  | Musculoskeletal and Connective Tissue Disorders | Pain In Extremity | 4 | 517 | 2 | 1998 | 7.73 (1.41 - 42.32) | 7.68 (7.76) |
|  | Nervous System Disorders | Dizziness* | 40 | 481 | 37 | 1963 | 4.41 (2.79 - 6.98) | 4.15 (47.4) |
|  |  | Hypoaesthesia | 3 | 518 | 1 | 1999 | 11.58 (1.2 - 111.53) | 11.52 (7.21) |
|  | Respiratory, Thoracic and Mediastinal Disorders | Nasal Congestion | 5 | 516 | 1 | 1999 | 19.37 (2.26 - 166.17) | 19.19 (14.41) |
|  | Vascular Disorders | Hypotension* | 9 | 512 | 5 | 1995 | 7.01 (2.34 - 21.02) | 6.91 (16.34) |
| ＞ 45 years | Ear and Labyrinth Disorders | Vertigo | 3 | 702 | 2 | 2891 | 6.18 (1.03 - 37.04) | 6.16 (5.19) |
|  | Gastrointestinal Disorders | Nausea | 18 | 687 | 24 | 2869 | 3.13 (1.69 - 5.8) | 3.08 (14.6) |
|  |  | Abdominal Pain Upper | 7 | 698 | 7 | 2886 | 4.13 (1.45 - 11.83) | 4.1 (8.25) |
|  |  | Abdominal Pain | 5 | 700 | 5 | 2888 | 4.13 (1.19 - 14.29) | 4.1 (5.88) |
|  | General Disorders and Administration Site Conditions | Fatigue* | 42 | 663 | 80 | 2813 | 2.23 (1.52 - 3.27) | 2.15 (17.63) |
|  |  | Feeling Abnormal | 14 | 691 | 9 | 2884 | 6.49 (2.8 - 15.06) | 6.38 (25.03) |
|  |  | Oedema Peripheral* | 13 | 692 | 15 | 2878 | 3.6 (1.71 - 7.61) | 3.56 (12.9) |
|  |  | Pain* | 7 | 698 | 7 | 2886 | 4.13 (1.45 - 11.83) | 4.1 (8.25) |
|  |  | Gait Disturbance | 4 | 701 | 3 | 2890 | 5.5 (1.23 - 24.62) | 5.47 (6.28) |
|  |  | Chills | 3 | 702 | 2 | 2891 | 6.18 (1.03 - 37.04) | 6.16 (5.19) |
|  | Investigations | Blood Pressure Decreased* | 19 | 686 | 8 | 2885 | 9.99 (4.35 - 22.91) | 9.75 (44.52) |
|  |  | Hepatic Enzyme Increased | 6 | 699 | 1 | 2892 | 24.82 (2.98 - 206.53) | 24.62 (19.46) |
|  |  | Blood Potassium Increased | 5 | 700 | 3 | 2890 | 6.88 (1.64 - 28.86) | 6.84 (9.37) |
|  |  | Blood Pressure Abnormal | 4 | 701 | 4 | 2889 | 4.12 (1.03 - 16.52) | 4.1 (4.7) |
|  |  | Heart Rate Decreased | 3 | 702 | 1 | 2892 | 12.36 (1.28 - 119) | 12.31 (7.8) |
|  | Musculoskeletal and Connective Tissue Disorders | Back Pain | 8 | 697 | 11 | 2882 | 3.01 (1.21 - 7.5) | 2.98 (6.14) |
|  | Nervous System Disorders | Dizziness* | 47 | 658 | 45 | 2848 | 4.52 (2.98 - 6.86) | 4.29 (59.44) |
|  |  | Somnolence | 13 | 692 | 14 | 2879 | 3.86 (1.81 - 8.26) | 3.81 (14.08) |
|  |  | Migraine | 5 | 700 | 3 | 2890 | 6.88 (1.64 - 28.86) | 6.84 (9.37) |
|  | Skin and Subcutaneous Tissue Disorders | Pruritus | 9 | 696 | 12 | 2881 | 3.1 (1.3 - 7.4) | 3.08 (7.26) |
|  |  | Night Sweats | 3 | 702 | 2 | 2891 | 6.18 (1.03 - 37.04) | 6.16 (5.19) |
|  | Vascular Disorders | Hypotension* | 14 | 691 | 3 | 2890 | 19.52 (5.59 - 68.1) | 19.15 (42.7) |
|  |  | Blood Pressure Fluctuation | 5 | 700 | 6 | 2887 | 3.44 (1.05 - 11.29) | 3.42 (4.68) |

**Supplementary Table 6** Data on preferred terms for individuals reporting episodes of loss of consciousness and theie indications.

| PRIMARYID | Indications | Preferred Terms |
| --- | --- | --- |
| 229176661 | IgAN | Dizziness |
|  |  | Drug Intolerance |
|  |  | Loss Of Consciousness |
| 229180812 | IgAN, CKD, Proteinuria | Nausea |
|  |  | Pain |
|  |  | Injury |
|  |  | Blood Pressure Decreased |
|  |  | Diarrhoea |
|  |  | Loss Of Consciousness |
|  |  | Muscle Spasms |
|  |  | Fall |
|  |  | Dysstasia |
|  |  | Dizziness |
|  |  | Limb Discomfort |
|  |  | Vomiting |
|  |  | Protein Total Decreased |
|  |  | Hypoaesthesia |
|  |  | Product Dose Omission Issue |
| 229180813 | IgAN, CKD, Proteinuria | Diarrhoea |
|  |  | Protein Total Decreased |
|  |  | Muscle Spasms |
|  |  | Nausea |
|  |  | Limb Discomfort |
|  |  | Pain |
|  |  | Fall |
|  |  | Blood Pressure Decreased |
|  |  | Loss Of Consciousness |
|  |  | Product Dose Omission Issue |
|  |  | Hypoaesthesia |
|  |  | Dizziness |
|  |  | Somnolence |
|  |  | Vomiting |
|  |  | Injury |
|  |  | Dysstasia |
